# Necrotizing soft tissue infection of both ear lobules occurring concomitantly in a set of twins following non-aseptic ear piercing: a case report

**DOI:** 10.1186/s12887-020-1952-2

**Published:** 2020-02-05

**Authors:** U. U. Nnadozie, O. B. Ezeanosike, C. C. Maduba, D. C. Obu, U. S. D. Unigwe

**Affiliations:** 1 Department of Surgery, Ebonyi State University, Abakaliki, Ebonyi State Nigeria; 2 Division of Plastic Surgery, Department of Surgery, Alex Ekwueme Federal University Teaching Hospital Abakaliki, PMB 102, Abakaliki, Ebonyi State 480001 Nigeria; 3 Newborn Special Care Unit, Department of Paediatrics, Alex Ekwueme Federal University Teaching Hospital Abakaliki, Abakaliki, Ebonyi State Nigeria; 4 Infectious Disease Unit, Department of medicine, University of Nigeria Teaching Hospital, Enugu, Nigeria

**Keywords:** Asepsis, Case-report, ear-piercing, Necrotizing soft-tissue infection

## Abstract

**Background:**

Necrotizing soft tissue infection of the ear following ear piercing is a very rare condition. It is easily misdiagnosed leading to reconstructive morbidities and mortality in neonates. High clinical suspicion is important for early diagnosis. Our knowledge, this is the first case reported in the literature in this unique initial presentation. We hope to heighten the awareness of necrotizing soft tissue infection of the ear following ear piercing to ensure early aggressive intervention.

**Case presentation:**

We report a set of 19-day-old female twin neonates who developed bilateral ear sores following ear piercing in a primary healthcentre without adherence to surgical asepsis. Examination findings showed features consistent with necrotizing soft tissue infections of the ears. They were successfully managed with antibiotics and wound care.

**Conclusion:**

Necrotizing soft tissue infections is a very rare complication of neonatal ear piercing. It may occur following suboptimal aseptic procedure and a high index of suspicion is necessary to make this diagnosis to ensure early intervention and to forestall the potential reconstructive morbidities and mortality associated with late recognition. Adherence to basic aseptic surgical principles is the key to prevention of necrotizing soft tissue infections.

## Background

Necrotizing soft tissue infection (NSTI) is a potentially life-threatening emergency that results from various combinations of organisms [1]. They are generally a rare condition that may affect any part of the body. Its rarity may contribute to the common misdiagnosis, which ranges between 41 and 96%.

in published reports [2]. It is often confused with cellulitis, abscess or other soft tissue infections and has been variously referred to as streptococcal gangrene, necrotizing erysipelas, hospital gangrene, suppurative fasciitis, and necrotizing fasciitis [3, 4]. The current nomenclature, NSTI, covers all diffuse necrotizing soft tissue infections other than gas gangrene [5].

NSTIs are known to affect most commonly the extremities, trunk and perineum [6]. Extremities account for over 50% in some reports and up to 70% in a study done in Northwest Nigeria [5–7]. They are associated with immune-suppression and poor nutritional state of the individual [7].

NSTIs are classified aetiologically and anatomically. Aetiologically, NSTIs are classified as polymicrobial or type 1, gram-positive monomicrobial or type 2, gram-negative monomicrobial or type 3 and fungal infection/ type 4. They are classified anatomically as cervicofacial, truncal, perineal or extremity [6, 7].

Cervico-facial NSTI is the rarest and often related to dental infections [6].It contributes only about 2.1% of all cases [7].It is associated with high morbidity and mortality due to proximity to airway and rapidity of progression [6].Case-fatality of over 60% has been reported [8]. It is, therefore, necessary to give it accelerated attention. In a case series, otitis media was a complication developed by a patient [8].

Necrotizing soft tissue infection involving the ear is very rare [9]. NSTIs affecting both ear lobules at the same time are much rarer. Interestingly, the infection occurred at the same time in both ears of a set of twins under a very rare circumstance too. We, therefore, highlight this set of twin neonates with concomitant bilateral ear lobule NSTI following unaseptic ear piercing in a primary health center.

## Case presentation

A set of female twin neonates was seen at the clinic as 19-day-old term babies delivered in primary health center. They had bilateral ear piercing without adherence to asepsis on the 9th day of life. No extreme pressure was applied to the ear during or after the procedure. The procedure was uneventful until 4 days after the procedure when the lobules were noticed to have swollen and darkened. There was no discharge from the wound, fever or refusal of feeds. There was no known history of atopy in the older siblings.

The mother removed the earrings on the fourth day after the ear piercing and noticed rims of demarcated dark tissues. Subsequently, the ears of the first twin developed abnormally large buttonholes instead of the earring pinhole. The second twin had some degree of distortion which necessitated presentation to a tertiary hospital.

**Fig. 1:**
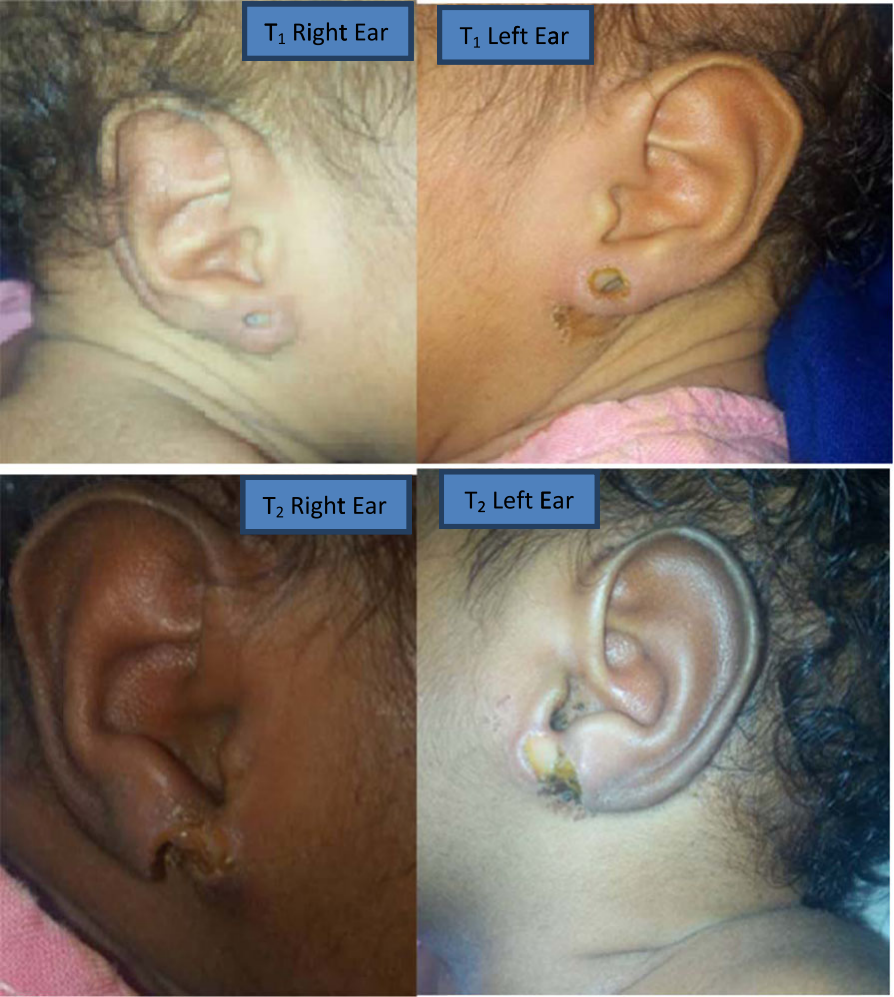
T_1_ right ear showed a buttonhole ulcer about 0.5 cm in the widest dimension with inflamed surrounding skin. T_2_ left ear showed a smaller buttonhole ulcer measuring 0.4 cm in the widest dimension. T_2_ right ear showed a 2 cm × 0.2 cm ulcer along the rim of the lobule with a necrotic bed and inflamed surrounding skin. T_2_ left ear had an ulcer measuring about 3 cm × 2 cm × 1 cm affecting whole thickness of the lobule with inflamed surrounding skin

Babies were delivered at 38 weeks of gestation without any preceding history of premature rupture of membrane or vaginal discharge in the mother. Babies were on exclusive breastfeeding. Examination revealed a 2.7 kg first twin (T_1_) with a temperature of 37.5 °C, pulse rate of 118 pm and respiratory rate of 56 cpm. The right ear showed a buttonhole ulcer about 0.5 cm in the widest dimension with inflamed surrounding skin. The left ear showed a smaller buttonhole ulcer measuring 0.4 cm in the widest dimension (Fig. 1).

The second twin (T_2_) weighed 3.7 kg with a temperature of 37.5 °C, pulse rate of 120 pulsations per minute and respiratory rate of 60 cpm. The right ear showed a 2cmx0.2 cm ulcer along the rim of the lobule with a necrotic bed and inflamed surrounding skin. The left ear had an ulcer measuring about 3cmx2cmx1cm affecting whole thickness of the lobule. The edge was undermined and the surrounding skin inflamed (Fig. 1).

A diagnosis of necrotizing soft tissue infection of both ears was made for each twin. They were admitted, wound swab and tissue specimen were sent for microscopy, culture and sensitivity (MCS) and histology respectively for the both twin. Erythromycin and metronidazole syrups were commenced empirically while awaiting sensitivity results. Wound care was provided by dressing wound with 5% povidone-iodine ointment.

T_1_ wound swab MCS showed growth of *Staphylococcus aureus* sensitive to erythromycin and ceftazidime but resistant to vancomycin and ampicillin, amoxicillin, and augmentin. T_2_ wound swab MCS yielded S*aureus* and *Pseudomonas aeruginosa* which were sensitive to ceftazidime, levofloxacin, augmentin but resistant to erythromycin.

After 2 days on admission, the parents declined further inpatient treatment on financial grounds and returned home on oral drugs. However, on telephone follow-up, the wounds were noted to have healed well and the twins remained healthy.

## Discussion

Necrotizing soft tissue infection of the ear is extremely rare with a tendency to misdiagnosis resulting in extensive tissue loss and concurrent reconstructive challenges [1, 10].In neonates it is even more likely to miss the diagnosis except with high clinical index of suspicion. It is predominantly an adult condition with a few cases found in neonates with omphalitis, mastitis, and post-operative wound infections. It has about 50% case-fatality in this age group [11]. The cervicofacial NSTI has a case-fatality of about 60% in the adult population and would probably be higher in neonatal age group [8].

Generally, paediatric skin and soft tissue infections are on the increase for suspicions of methicillin-resistant *S. aureus* [12]. Streptococcus and staphylococcus are the major organisms incriminated in paediatric infections. Streptococcus is a leading cause of type 2 NSTI [13]. In our report, T_1_ yielded only *S.aureus*, while T_2_ yielded both *S.aureus* and *P.aeroginosa*. The first twin is a type 2 disease while the second twin is a type 1 disease. A similar case report in a neonate with type 2 chest NSTI yielded only *S.aureus* [14].

In most cases of neonatal NSTI, there are known predisposing conditions such as anaemia, malnutrition, diabetic mother, hyperbilirubinemia and other immunosuppressive conditions.^13^In our report, only failure to adhere to surgical asepsis was incriminated.

Ear piercing is an age-long practice in different cultures done for different reasons. It could be done by both professional and non-professional health attendants depending on the setting. It is also associated with different types of complications of which infection is one [15].

The organisms gain entrance either through inoculation such as through ear piercing which introduces them into the tissue or through a haematogenous spread [13]. In this report it is suspected to have resulted from inoculation during the ear piercing. Since this is the most likely route of infection for our patients, the need for strict adherence to surgical asepsis during ear-piercing is emphasized in this report.

The patients were placed on appropriate antibiotics. They recovered with antibiotic therapy and wound dressing with 5% povidone-iodine ointment. Follow up continued by telephone because of financial constraints and the patients may present for reconstruction depending on availability of funds.

Necrotizing soft tissue infections of the ear occurring concomitantly in a set of twins is very rare. It is potentially fatal and can be misdiagnosed in neonates. It is an uncommon complication of ear piercing in neonates and therefore not readily suspected. A high index of suspicion is crucial to early recognition and intervention forestalling potential challenging reconstructive morbidities and mortality. Adherence to basic aseptic surgical principles is the key to its prevention.. We therefore aim to present this case report so as to heighten awareness of earlobe NSTIs to give early aggressive attention, reduce cosmetic and reconstructive challenges and improve survival.

## Data Availability

Not applicable.

## Abbreviations

MCS: Microscopy, culture and sensitivity
NSTI: Necrotizing soft tissue infection
